# Differential Roles of Oxytocin Receptors in the Prefrontal Cortex and Nucleus Accumbens on Cocaine Self-Administration and Reinstatement of Cued Cocaine Seeking in Male Rats

**DOI:** 10.1093/ijnp/pyad059

**Published:** 2023-10-24

**Authors:** Rachel D Penrod, Makoto Taniguchi, Angela M Kearns, Jordan L Hopkins, Carmela M Reichel

**Affiliations:** Department of Neuroscience, Medical University of South Carolina, Charleston, South Carolina, USA; Department of Neuroscience, Medical University of South Carolina, Charleston, South Carolina, USA; Department of Neuroscience, Medical University of South Carolina, Charleston, South Carolina, USA; Department of Neuroscience, Medical University of South Carolina, Charleston, South Carolina, USA; Department of Neuroscience, Medical University of South Carolina, Charleston, South Carolina, USA

**Keywords:** Oxytocin receptor, addiction, relapse, shRNA

## Abstract

**Background:**

Little is known about the specific roles of cortical and accumbal oxytocin receptors in drug use disorders. To better understand the importance of the endogenous oxytocin system in cocaine relapse behavior, we developed an adeno-associated viral vector–expressing short hairpin (sh) RNAs to selectively degrade the rat oxytocin receptor (OxyR) mRNA in vivo.

**Methods:**

Male (Sprague-Dawley) rats received bilateral infusions of the shRNA for the oxytocin receptor (shOxyR) or an shRNA control virus into the prefrontal cortex (PFC) or the nucleus accumbens core (NAc). Rats self-administered cocaine on an escalating FR ratio for 14 days, lever responding was extinguished, and rats were tested for cued and cocaine-primed reinstatement of drug seeking.

**Results:**

OxyR knockdown in the PFC delayed the acquisition of lever pressing on an fixed ratio 1 schedule of reinforcement. All rats eventually acquired the same level of lever pressing and discrimination, and there were no differences in extinction. OxyR knockdown in the NAc had no effect during acquisition. In both the PFC and NAc, the shOxyR decreased cued reinstatement relative to shRNA control virus but was without effect during drug-primed reinstatement. OxyR knockdown in the PFC increased chamber activity during a social interaction task.

**Conclusions:**

This study provides critical new information about how endogenous OxyRs function to affect drug seeking in response to different precipitators of relapse. The tool developed to knockdown OxyRs in rat could provide important new insights that aid development of oxytocin-based therapeutics to reduce return-to-use episodes in people with substance use disorder and other neuropsychiatric disorders.

**Significance Statement**

Oxytocin is a neuroactive peptide that is being considered as a therapeutic for drug use disorder. In animal models, exogenous oxytocin delivery decreases drug seeking; however, very little research has focused on the endogenous oxytocin system during drug taking or seeking. Here, we developed and used a gene interference strategy to selectively degrade oxytocin receptors in targeted brain areas associated with drug use disorder. Developing tools to study the endogenous oxytocin function will lead to a better understanding of the system.

## INTRODUCTION

Cocaine use disorder continues to be a world health problem without an effective form of pharmacotherapy despite the groundbreaking strides that have been made in understanding the neurobiology of addiction. Recently, oxytocin (OXY) has received increased interest as a treatment for many neuropsychiatric disorders, including cocaine use disorder. In fact, clinical trials indicate that OXY is already being used therapeutically for substance use disorders. Neuroimaging studies (functional MRI) show that in response to drug-conditioned cues, intranasal OXY decreases neural activity in the dorsal medial prefrontal cortex (PFC) and modulates cortical-striatal connectivity during presentation of drug-associated cues (Bach et al., 2019; Joseph et al., 2019a, 2019b). Research defining the role of OXY in cocaine use disorder as well as an understanding of its mechanisms will advance our knowledge of OXY’s use as a therapeutic strategy for addiction.

OXY is a well-characterized neuroendocrine hormone produced within the paraventricular nucleus and supraoptic nucleus of the hypothalamus. OXY cells project to several areas involved in addiction, including cortical, limbic regions, and striatal areas (Knobloch and Grinevich, 2014). Oxytocin receptors (OxyRs) are ubiquitous throughout the brain, are Gq-coupled, and activate transduction pathways that include IP3 receptor activation and the release of intracellular calcium stores (Gimpl and Fahrenholz, 2001). More specifically, the paraventricular nucleus has axonal projections to the prefrontal cortex (PFC) (reviewed in Bowen and Neumann, 2017), and OxyRs are on PFC glutamatergic pyramidal neurons that project to the NAc (Tan et al., 2019); these areas are crucial nuclei in the drug relapse circuit, and OXY in each of these areas uniquely affects drug seeking. In the nucleus accumbens core (NAc), OxyRs are localized on parvalbumin (PARV+) interneurons (Dolen et al., 2013) and astrocytes (Moussawi et al., 2011; Dolen et al., 2013), and they form dopamine receptor D2 (D2R)/OxyR heteromers in D2R-expressing neurons upon activation (Bowen and Neumann, 2017). Additionally, they are expressed on serotonergic neurons projecting from the dorsal raphe nucleus to the NAc [the involvement of the serotonergic system in addiction is reviewed in (Cunningham and Anastasio, 2014)]. As such, OxyRs are ubiquitous and affect multiple neurotransmitter systems to maintain brain homeostasis. The endogenous OXY system undergoes adaptive changes following psychostimulant exposure. For example, OxyR density in the rodent amygdala and hypothalamus increases following methamphetamine, cocaine, and nicotine (Zanos et al., 2014, 2015; Georgiou et al., 2016a, 2016b), whereas methamphetamine also decreased OxyR immunoreactive fibers in the NAc (Baracz and Cornish, 2016). These findings position OXY as a critical regulator of addiction-related circuit plasticity.

To date, most addiction research testing the efficacy of OXY as a pharmaceutical approach has focused on exogenous OXY administration through peripheral or central delivery systems (Baracz and Cornish, 2016; Leong et al., 2018). Our laboratory (among others) has shown that systemic OXY administration decreases cued reinstatement of methamphetamine (Cox et al., 2013, 2017; Bernheim et al., 2017), heroin (Leong et al., 2018; Carter et al., 2023), and cocaine (Leong et al., 2016, 2017; Weber et al., 2018) seeking in male and female rats. More recently, we discovered that site-specific application of OXY into the prelimbic area of the PFC increased cocaine-cued reinstatement (Weber et al., 2018). This increase in drug seeking is specific to cocaine’s effects in the PFC because intracerebroventricular, NAc, and subthalamic nucleus OXY administration decreased cued reinstatement of cocaine seeking (Leong et al., 2017, 2018). Notably these described decreases also occur with methamphetamine (Baracz and Cornish, 2016).

Very little research has investigated how the endogenous OXY system may modulate cued reinstatement, largely because we are limited by the molecular tools available to study the endogenous system. Here, we assess the specific contribution of the endogenous system with use of an adeno-associated viral vector expressing a short hairpin RNA (shRNA), developed in our laboratories, that selectively degrades the endogenous rat OxyR mRNA in infected cells. Given the well-documented role of OXY in mediating responses to social stimuli (Froemke and Young, 2021) and OXY’s affects on shared drug- and social-reward circuitry (Insel, 2003; Buisman-Pijlman et al., 2014), we conducted a social interaction test following reinstatement of cocaine seeking to explore an additional behavior modulated by OXY.

## METHODS

### Subjects

A total of 46 male Sprague-Dawley rats (Harlan, Indianapolis, IN, USA) were used in this experiment. All rats weighed 250–300 g at the time of arrival. Rats were single-housed on a reversed 12-hour-light/-dark cycle (lights off at 6:00 am) in a temperature- and humidity-controlled vivarium. Single housing was used to prevent partners from chewing on their catheter ports. All experimental procedures were conducted during the dark cycle. Rats received water ad libitum and were kept on a stable intake diet 20–40 g of standard rat chow daily throughout the study. Procedures were conducted in accordance with the “Guide for the Care and Use of Laboratory Rats” (Institute of Laboratory Animal Resources on Life Sciences, National Research Council, 2011).

### Development of an shRNA for the Rat OxyR

**Figure 1.**
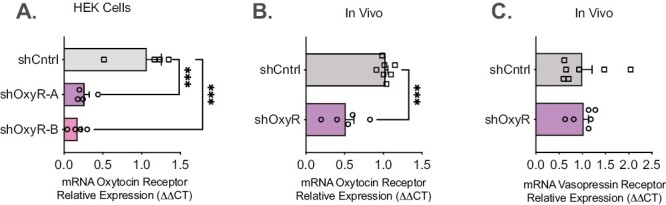
Development and validation of the short hairpin Ribonucleic acid (RNA) for the oxytocin receptors (OxyR). (A) We generated 2 shRNA sequences targeting rat OxyR mRNA but not the vasopressin receptor messenger (m)RNA. Both sequences significantly knocked down overexpressed OxyR in human embryonic kidney (HEK) cells. (B) The shRNA for the OxyR significantly reduced OxyR mRNA in vivo using brain tissue. (C) The shRNA did not affect vasopressin receptor mRNA. ***P < .0001.

We generated 2 shRNA sequences targeting rat OxyR mRNA but not the vasopressin receptor mRNA using Integrated DNA Technologies (IDT, Coralville, IA, USA). The targeted RNAi sequences were GGATCACGCTCGCCGTCTA (termed shOxyR-A) and GCTCGCCGTCTACATTGTA (termed shOxyR-B), ensuring that these sequences are not conserved in other mRNA (with no more than 15 bp). pAAV2-shOxyR-eGFP (and shLuciferase) were constructed by the generation, in vitro oligomerization, and phosphorylation of custom DNA oligos (IDT) directed against OxyR or Luciferase. pAAV2-shRNA-eGFP was digested with *Xba*I and *Spa*I, and custom oligos were ligated into the dephosphorylated vector. Following subcloning into the pAAV-shRNA-EGFP plasmid, we used an overexpression strategy for the OxyR (pCMV-OXTR; RR204321) to validate the effect of OxyR shRNA in vitro (see Figure 1A). HEK293 (ATCC: CRL-3216) cells were transfected with the rat OxyR overexpression vector with shRNA Luciferase (shCntrl) or OxyR shRNA (shOxyR-A and B) in quadruple. The extent of shRNA-mediated knockdown was quantified with quantitative real time PCR (qPCR). Our shOxyR-A and B robustly reduced the OxyR mRNA expression compared with shCntrl. Subsequently, following sequence validation and endotoxin-free maxi-prep (Qiagen), plasmid DNA was packaged into AAV2 and purified through the UNC Vector Core for in vivo use. To test our construct, we infused 1 µL of high titer (10^12^ vg/mL or greater) AAV2-shOxyR or AAV2-shCntrl into the PL area of the mPFC. Following at least 4 weeks of viral expression, rats were killed, and slices of the PL were observed under an epifluorescent microscope (Nikon Eclipse 80i). EGFP-positive areas of the target brain regions were excised and processed for qPCR analysis.

### Quantitative Real-Time PCR Taniguchi et al., 2017;Penrod et al., 2018

mRNA expression was assessed via qPCR as described in previous work (**Taniguchi et al., 2017; Penrod et al., 2018)**. Using specific primers for OXY (OxyR, forward primer, TGCCCCAGTCTCGCTTGCT; reverse primer, TCCAGGTCTAGCGCAGCCC); calnexin (calnexin, forward primer, GCTCTGGTCCATGACATCCG; reverse primer, CAGCATCTGCCCCACTACAC); vasopressin receptor, forward primer, GCTCAACACTACGCTCTCTGCTTG; reverse primer, GTCTCAGCTCCATGTCGGATGT. OxyR mRNA expression, normalized to calnexin (in vivo), was calculated using the ΔΔCt method (Pfaffl, 2001) and expressed as fold change in relation to control groups.

### Surgery

Rats were anesthetized with i.p. injections of ketamine (66 mg/kg, i.p., Vedco Inc, St Joseph, MO, USA) mixed with xylazine (1.3 mg/kg, i.p., Lloyd Laboratories, Shenandoah, IA, USA) and equithesin (0.5 mL/kg). Ketorolac (2.0 mg/kg, i.p., Sigma, St. Louis, MO, USA) was given before stereotaxic and jugular cannulation surgery as an analgesic. Surgical procedures were conducted using aseptic techniques. Rats received bi-lateral infusions of shOxyR or shCntrl (1 µL) into the PFC or NAc. The rat was placed in the stereotactic frame (Kopf), and the skin overlying the skull was cut open. The skull was cleared, and bregma and lambda were positioned at the same dorsal-ventral (DV) coordinate. We targeted the PL area of PFC using the following coordinates: AP +2.8 mm, ML* ± *0.6 mm, and DV −2.7 mm measured from bregma. We targeted the NAc core with the following coordinates: AP +1.6 mm; ML* ± *2.8 mm (10° angle); DV −7.1 mm. Jugular catheters were implanted under the same plane of anesthesia. One end of a silastic catheter was implanted into the external right jugular vein. The other end ran subcutaneously, exited from a small incision on the back, and attached to an infusion cannula (PlasticsOne Inc., Roanoke, VA, USA). Cephazolin (10 mg/0.1 mL) was given postsurgery (0.1 mL i.v.) and during recovery along with 0.05 mL of taurolidine-citrate catheter solution (Access Technologies, Skokie, IL, USA).

### Cocaine Self-Administration, Extinction, and Reinstatement

All self-administration experiments were conducted during the rats’ dark cycle in standard Plexiglas self-administration chambers (30 × 20 × 20 cm) that were enclosed in sound attenuating cubicles with a ventilation fan (Med Associates, St. Albans, VT, USA) and linked to a computerized data collection program (MED PC, Med Associates). Each chamber was equipped with 2 retractable levers with a white stimulus light above each lever, a house light, and a tone generator. For cocaine self-administration, infusion tubing was enclosed in steel spring leashes (Plastics One Inc) and connected to the infusion cannula and a weighted swivel apparatus (Instech, Plymouth Meeting, PA, USA) that was suspended above the box to allow for free movement within the chamber.

Cocaine hydrochloride (provided by the National Institute on Drug Abuse, Research Triangle Park, NC, USA) was dissolved in 0.9% sterile saline and administered at a dose of 0.5 mg/kg/infusion. Self-administration sessions were conducted 6 d/wk for 2 h/d. The house light remained on throughout the sessions, and a response on the active lever resulted in activation of the pump and delivery of a 2-second cocaine infusion and a 5-second presentation of a stimulus complex (illumination of the white stimulus light over the active lever and activation of tone generator; 78 dB, 4.5 kHz), followed by a 20-second time-out. During the time-out period, responses on the active and inactive levers were recorded but had no scheduled consequences. Rats initially self-administered cocaine along a fixed ratio 1 schedule of reinforcement (1 lever press resulted in a drug infusion) until they reached the criterion of a minimum of 5 days with >10 infusions. Rats then moved to a FR3 schedule for a minimum of 3 days, followed by a FR5 schedule for the remainder of the self-administration sessions. To verify catheter patency when rats were not administering cocaine, a 0.10- to 0.12-mL infusion of methohexital sodium (Eli Lilly, Indianapolis, IN, USA), a short-acting barbiturate that produces a rapid loss of muscle tone when administered intravenously. After each self-administration session, rats’ catheters were flushed with 0.05 mL taurolidine-citrate catheter solution.

Following self-administration, rats underwent 2-hour daily extinction sessions for a minimum of 7 days, where responses on both the active and inactive levers were recorded but had no scheduled consequences. Extinction criterion consisted of <25 active lever presses for 3 consecutive days. Upon reaching extinction criteria, rats underwent cued reinstatement tests for 2 hours on a fixed ratio 1 schedule of reinforcement. During cued reinstatement, responses on the active lever resulted in presentation of the light + tone stimulus complex, but no drug was delivered. During drug primed reinstatement rats receive a 10-mg/kg, i.p. cocaine injection before chamber placement. Responses on the active lever were without scheduled consequence, and responses on the inactive lever were also recorded on all tests.

### Social Interaction

Social interaction tasks assess approach/avoidance behaviors in response to an unfamiliar rat (Carter et al., 2020, 2023; Cox et al., 2022). The ratio of time spent with and without the social partner can be taken as an index of anxiety-like behavior (Lezak et al., 2017). These tasks also assess social reward as an index of basic reward processing. Following reinstatement testing rats underwent social interaction tasks in a round open field (125-cm diameter), and behavior was recoded with Ethovision tracking software in 10-minute sessions. On day 1 rats were placed in the open field and given 10 minutes to explore the environment; this is considered a pre-interaction measure. White tape was used to mark a 11- × 7-inch square on the floor of the apparatus to indicate the area where the caged rats would go on subsequent sessions. The amount of time spent in this area was recorded. On days 2, 3, and 4 an unfamiliar rat was placed in a wire cage [11 × 7 × 7, (*l × w × h*)] in a marked section of the open field. During these sessions, we evaluated the amount of time rats spent in the same zone at the caged rat, approaches to the rat, and chamber activity for a 10-minute session. On day 5, rats were returned to the empty open field for a post-interaction assessment and time spent in the area that previously held the conspecific (denoted by the white tape) was recorded.

### Statistical Analysis

Statistical analyses were conducted with mixed variable ANOVA with appropriate planned comparisons and post hocs. The main independent variables consisted of test (ext or test) as a within-subjects and group (shCntrl vs shOxyR) as between-subjects variables. Lever (active vs inactive) and day (when analyzing self-administration data) were additional within-subjects variables depending on the experiment. Statistics were carried out using Prism 10 statistical software. Planned comparisons between Groups (shCntrl and shOxyR) on test days and post hoc comparisons were conducted with a Holm’s Sidak correction. Data is be represented as means ± SEM, with the alpha set at *P < *.05.

## Results

### Knockdown of the OxyR in Vitro

Figure 1A shows the extent of shRNA knockdown in vitro quantified with qPCR. When co-transfected into HEK293 cells, our shOxyR-A and B robustly reduced overexpressed rat OxyR mRNA compared with shCntrl (1-way ANOVA, F_(2,9)_ = 17.35, *P* = .0008, post hoc *P < *.05). Following successful packaging of shOxyR-A into an AAV2 (referred to as shOxyR in all further figures), Figure 1B shows successful knockdown of endogenous OxyR mRNA in AAV2-shOxyR infected areas of the PFC (t_(10)_ = 5.48, *P* = .001). Because the homology between the OXY and vasopressin receptor molecules is high, we specifically designed shRNA sequences against nonconserved regions. Figure 1C shows specificity of our shOxyR to OxyR rather than vasopressin receptor in the PFC (t_(11)_ = 0.12, *P* = .4533).

### Experiment 1: ShOxyR Knockdown in the PFC

#### Acquisition of Cocaine Self-Administration—

**Figure 2.**
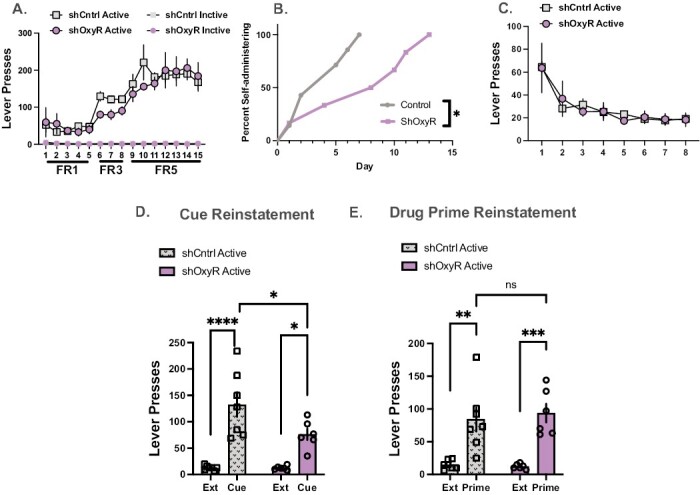
The impact of short hairpin oxytocin receptor (shOxyR) in the prefrontal cortex (PFC) on cocaine taking and seeking. (A) There were no differences in active or inactive lever responding between sh control (Cntrl) and shOxyR rats. (B) Relative to control rats, shOxyR delayed acquisition of the operant response. The control rats reached 100% acquisition in 7 days, whereas the shOxyR did not reach 100% criteria until day 13. (C) There were no differences in extinction responding between shCntrl and shOxyR rats. (D) During cued reinstatement, both shCntrl and shOxyR increased responding relative to extinction. Control rats had higher reinstatement relative to shOxyR. (E) On the drug primed test, both groups reinstated relative to extinction. *P < .05, **P < .0015, ***P < .009, ****P < .0001.

All rats readily acquired cocaine self-administration and discriminated active vs inactive levers (Figure 2A). Data were analyzed with a mixed 3-way (day × lever × group) ANOVA. There was a day main effect (F_(14,313)_ = 15.45, *P < *.0001; lever main effect, F_(1,313)_ = 789.2, *P < *.0001) and a day × lever interaction (F_(14,313)_ = 15.55, *P < *.0001). The group main effect was not significant (F_(3,313)_ = 1.9, *P* = .16) and did not interact with day (F_(14,313)_ = 0.68, *P* = .78) or lever (F_(3,313)_ = 0.17, *P* = .19). Also, the 3-way interaction was not significant (F_(14,313)_ = 0.67, *P* = .79). However, shOxyR delayed acquisition of cocaine self-administration as indicated by the number of days it took for 100% of the cohort to reach self-administration criterion, defined as earning a minimum of 10 infusions during the 2-hour session (Figure 2B), compared with shCntrl animals (Log-rank Mantel-Cox test chi^2^ = 4.16, *P* = .041). All the control rats were at performance criteria by day 7 of cocaine self-administration, whereas the entire group of shOxyR rats did not reach criteria until day 13.

#### Extinction and Reinstatement—

All rats extinguished responding on the active lever (Figure 2C). The 2-way mixed ANOVA revealed a day main effect, F_(7,73)_ = 16.55, *P < *.0001. The main effect of group was not significant (F_(1,11)_ = 0.04, *P* = .84), and group did not interact with day (F_(7,73)_ = 0.54, *P* = .8). On the cued reinstatement test, shOxyR knockdown decreased lever pressing relative to the control group (Figure 2D). Results from the test × group ANOVA revealed that both groups reinstated drug seeking relative to extinction (Figure 2D, main effect of test, F_(1,22)_ = 44.15, *P < *.0001). The test × group interaction approached significance (F_(1,22)_ = 4.12, *P* = .055) as did the group main effect (F_(1,22)_ = 4.27, *P* = .051). ShCntrl rats had more active lever presses relative to shOxyR rats (Holm Sidak, *P < *.05). On the drug-primed reinstatement test, shOxyR knockdown did not decrease responding between Control and shOxyR rats (Figure 2E). Results from the ANOVA reveal that both groups reinstated drug seeking relative to extinction (Figure 2E, main effect of test, F_(1,22)_ = 38.46, *P < *.0001). The main effect of group (F_(1,22)_ = 0.08, *P* = .77) and test × group interaction (F_(1,22)_ = 25, *P* = .77) were not significant. There were no significant differences on inactive lever responding on either test; means and standard errors are presented in Table 1.

**Table 1.** Inactive lever responding on tests (mean* ± *SE)

Experiment	Reinstatement test	Control	shOxyR
Experiment 2: PFC	Cue	8.71* ± *2.76	5.33* ± *1.93
	Prime	1.14* ± *0.40	2.17* ± *0.83
Experiment 4: NAc	Cue	5.86* ± *1.48	8.12* ± *2.39
	Prime	3.57* ± *1.36	6.12* ± *2.13

Abbreviations: NAc, nucleus accumbens core; PFC, prefrontal cortex; shOxyR, short hairpin RNA for the oxytocin receptor.

#### Social Interaction—

**Figure 3.**
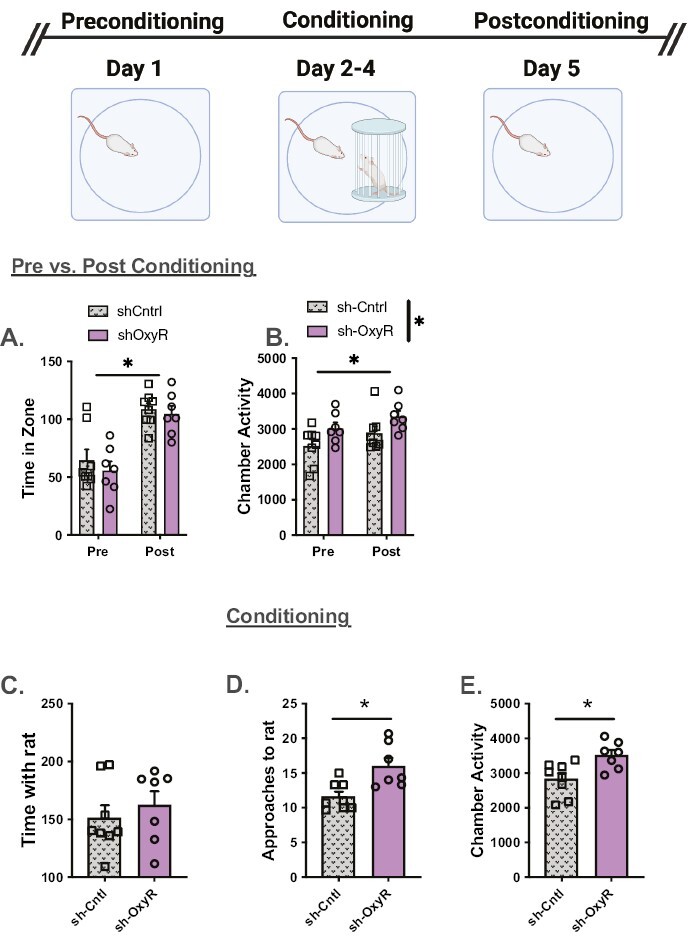
The impact of shOxyR in the PFC on social behaviors. (A) shCntrl and shOxyR spent significantly more time in an area previously associated with the presences of a conspecific on the posttest relative to the pretest. (B) Both groups of rats were more active on the posttest, and shOxyR rats were more active than shCntrls. (C) There were no differences in time spent with the conspecific during conditioning. (D) Control rats had fewer approaches to the conspecific compared with shOxyR rats, and (E) shOxyR rats were more active during the test. *P < .05.

On the social interaction task both groups displayed a preference for the area in the apparatus associated with a conspecific (Figure 3A). The 2-way mixed test × group ANOVA revealed a main effect of test (pre- vs postsocial interaction) (F_(1,13)_ = 33.61, *P < *.0001). The group main effect (F_(1,13)_ = 0.9, *P* = .36) and the group × test interaction (F_(1,13)_ = 0.1, *P* = .76) were not significant. Chamber activity recorded during the pre and post sessions were higher on the post test and in shOxyR rats (Figure 3B). Specifically, the results from the ANOVA revealed a main effect of test (F_(1,13)_ = 6.4, *P* = .025) and a main effect of group (F_(1,13)_ = 5.44, *P* = .037). The test × group interaction was not significant (F_(1,13)_ = 0.16, *P* = .9). During the conditioning sessions, shCntrl and shOxyR rats spent the same amount of time with the conspecific (Figure 3C, t_(13)_ = 0.69, *P* = .25), but the shOxyR rats had more approaches to the caged rats (Figure 3D, t_(13)_ = 3.27, *P < *.003) and were more active overall during the sessions (Figure 3E; t_(13)_ = 2.97, *P < *.005) relative to shCntrl rats.

### Experiment 2: Knockdown of shOxyR in the NAc

#### Acquisition of Cocaine Self-Administration—

**Figure 4.**
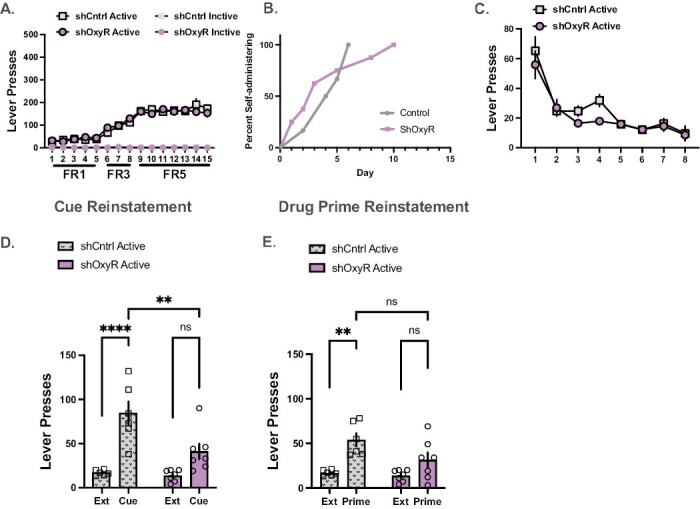
The impact of short hairpin oxytocin receptor (shOxyR) in the nucleus accumbens core (NAc) on cocaine taking and seeking. (A) There were no differences in active or inactive lever responding between control and shOxyR rats. (B) There were no differences in days to acquire the operant response. (C) There were no differences in extinction responding between shCntrl and shOxyR rats. (D) During cued reinstatement, control rats increased responding relative to extinction and had higher reinstatement relative to shOxyR. (E) On the drug primed test, only control rats reinstated above extinction. **P < .0015, ****P < .0001.

All rats readily acquired self-administration and discriminated active vs inactive levers (Figure 4A). Data were analyzed with a mixed 3-way (day × lever × group) ANOVA with day and lever as within subjects variables and group (shOxyR or shCntrl) as between subjects. There was a day main effect, F_(14,357)_ = 46.88, *P < *.0001; lever main effect, F_(1,357)_ = 2310, *P < *.0001; and a day × lever interaction, F_(14,357)_ = 46.95, *P < *.0001). The group main effect was not significant (F_(3,357)_ = 0.92, *P* = .92) and did not interact with day (F_(14,357)_ = 0.776, *P* = .7) or lever (F_(3,357)_ = 0.12, *P* = .73). Also, the 3-way interaction was not significant (F_(14,357)_ = 0.72, *P* = .75). There were no differences in the number of days required to reach self-administration criterion, defined as earning a minimum of 10 infusions during the 2-hour session (Figure 4B). All rats reached criterion between 4 and 5 days.

#### Extinction and Reinstatement—

All rats extinguished responding on the active lever (Figure 4C). The 2-way mixed ANOVA revealed a day main effect, F_(7,49)_ = 30.21, *P < *.0001. The main effect of group was not significant (F_(1,7)_ = 0.45, *P* = .52), and group did not interact with day (F_(7, 27)_ = 0.98, *P* = .47). On the cued reinstatement test, shOxyR knockdown decreased lever pressing relative to the shCntrl group (Figure 4D). The test × group interaction was significance (F_(1,22)_ = 6.17, *P* = .02) as were the test (F_(1, 22)_ = 34.44, *P < *.0001) and group main effects (F_(1,22)_ = 8.24, *P* = .009). Active lever presses were significantly elevated in shCntrl rats in response to the cue relative to extinction responding and shOxyR rats (Holm Sidak, *P < *.05). On the drug-primed reinstatement test, there was a main effects of test (F_(1,22)_ = 20.94, *P* = .0001) and group (F_(1,22)_ = 4.59, *P* = .044), but the interaction was not significant (F_(1,22)_ = 2.63, *P* = .12) (Figure 4E). Lever responding increased on a cocaine prime in shCntrl rats relative to extinction, but the shOxyR rats did not respond above extinction levels (Holm’s Sidak, *P < *.05). There were no significant differences on inactive lever responding on either test. Means and standard errors are presented in Table 1.

#### Social Interaction—

**Figure 5.**
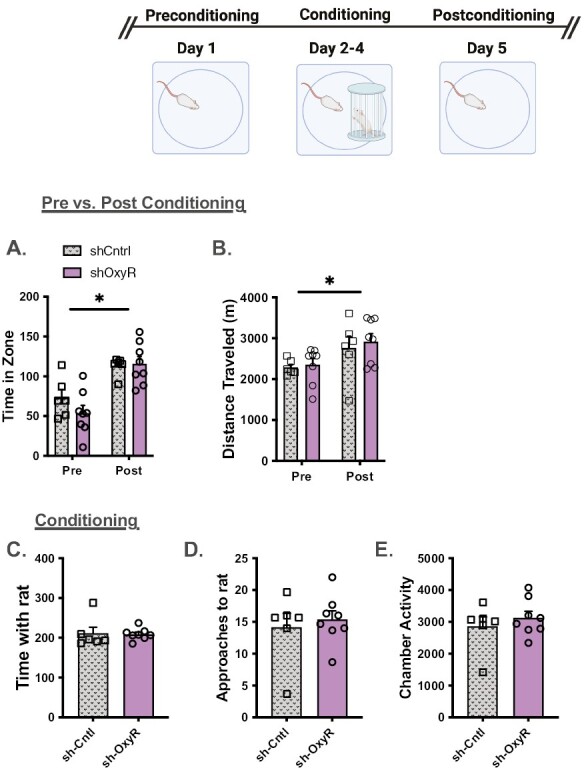
The impact of shOxyR in the NAc on social behaviors. (A) Control and shOxyR spent significantly more time in an area previously associated with the presences of a conspecific on the posttest relative to the pretest. (B) Both groups of rats were more active on the posttest. (C) There were no differences in time spent with the conspecific during conditioning. (D) Approaches to the conspecific; and (E) activity during the test. *P < .05.

On the social interaction task both groups displayed a preference for the area in the apparatus associated with a conspecific (Figure 5A). The 2-way mixed test × group ANOVA revealed a main effect of test (pre- vs postsocial interaction) (F_(1,1)_ = 26.04, *P < *.0003). The group main effect (F_(1,12)_ = 1.05, *P* = .32) and the group × test interaction (F_(1,12)_ = 1.2, *P* = .3) were not significant. Chamber activity recorded during the pre- and post sessions were higher on the post test for both groups (Figure 5B). Specifically, the results from the ANOVA revealed a main effect of test (F_(1,12)_ = 10.75, *P* = .007). The main effect of group (F_(1,12)_ = 0.27, *P* = .61) and the test × group interaction (F_(1,12)_ = 0.08, *P* = .77) were not significant. During the conditioning sessions, shRNA knockdown of the OxyR in the NAc did not affect time with the rat (Figure 5C, t_(12)_ = 0.118, *P* = .45), approaches (Figure 5D; t_(12)_ = 0.13, *P* = .31), or chamber activity (Figure 5E; t_(12)_ = 0.79, *P* = .22).

## DISCUSSION

Overall, our pattern of findings suggests different roles for PFC and NAc OxyRs in acquisition of cocaine self-administration, cocaine taking, reinstatement, and social behaviors (see Table 2). These findings result from shRNA interference of the OxyR in these brain areas. The novel shRNA constructs used in this study allow for interrogation of endogenous OxyR activity in the presence or absence of exogenously administrated oxytocin. After identifying 2 shRNA target sequences with high specificity for the OxyR (and no homology to the vasopressin receptor), both shRNA sequences were subcloned into an AAV-shRNA vector and tested for knockdown efficiency against overexpressed rat OxyR mRNA. Despite the high level of OxyR mRNA expression produced by the overexpression vector, both sequences produced significant knockdown compared with the shCntrl sequence (Figure 1). We opted to package the shOxyR-A sequence into AAV2 for in vivo administration. As expected, based on in vitro findings, AAV2-shOxyR robustly reduced endogenous OxyR expression without affecting the closely related vasopressin receptor.

**Table 2.** Summary of shOxyR knockdown effects

Brain area	Group	Self-administration	Reinstatement	Social interaction
PFC	Control	Standard acquisition curve	Above extinction and shOxyR	Typical time, approaches, and activity
	shOxyR	Delayed acquisition	Above extinction below Control	More approaches and activity relative to control
NAc	Control	Standard acquisition curve	Above extinction and shOxyR	Typical time, approaches, and activity
	shOxyR	Standard acquisition curve	Not above extinction and below control	Typical time, approaches, and activity

Abbreviations: NAc, nucleus accumbens core; PFC, prefrontal cortex; shOxyR, short hairpin RNA for the oxytocin receptor.

First, we report that shRNA knockdown of the OxyR in the PFC delayed acquisition of cocaine self-administration without an effect on cocaine intake. The same knockdown in the NAc was without effect on acquisition. Second, shOxyR in the PFC and NAc decreased responding to a cocaine cue but was without an effect on drug-primed reinstatement. Interestingly, NAc shOxyR prevented reinstatement responding defined by significant lever pressing above extinction values. Finally, shOxyR in the PFC increased chamber activity but not time spent with a caged conspecific in a social interaction task. In contrast, NAc shOxyR did not influence any measure of social interaction. A limitation of this report is the use of a male sample, so any extrapolation to females merits caution. However, we have reported sex similarities in past studies. As an exemplar, site-specific application of OXY in the PFC and NAc increased and decreased, respectively, cued reinstatement in both male and female rats (Weber et al., 2018). Further, in both sexes, systemic oxytocin administration decreased PFC and NAc c-fos levels in response to a cocaine associated cue (Leong et al., 2017).

We chose OxyR knockdown in the PFC and NAc because of our previous finding of opposing effects of site-specific OXY infusions into these areas on cued reinstatement tests (Weber et al., 2018). In those studies, rats underwent the same cocaine self-administration, extinction, and reinstatement procedures described in the current report. On cue reinstatement test day, OXY was bilaterally infused into the PFC or the NAc. We hypothesized that administration of supraphysiological levels of OXY in the PFC increased cued reinstatement via activation of OxyRs on glutamatergic neurons that project to the NAc. As such, in the PFC, OXY acts on its receptor to modulate glutamate release in the NAc. Our shOxyR resulted in an approximate 50% decrease in OxyR availability in the PFC, thereby decreasing endogenous PFC OXY signaling that would correspondingly decrease cued reinstatement, presumably through a reduction of glutamate release into the NAc (Logan et al., 2022). The PFC glutamatergic innervation of the NAc is a central pathway involved in relapse [reviewed recently in (Koob and Volkow, 2016)], and pharmacological inactivation of the PL or optogenetic inhibition of cortical projection neurons to the NAc prevents reinstated cocaine seeking (McLaughlin and See, 2003; Di Pietro et al., 2006; Stefanik et al., 2016). Cell type–specific knockdown of OxyRs on glutamatergic pyramidal cells projecting to the NAc is beyond the scope of this report but is the logical progression for this research.

In the NAc, there are multiple ways that OXY may act on its receptors to decrease drug seeking, including activation of OxyRs localized on parvalbumin (PARV+) interneurons (Dolen et al., 2013), activation of D2R-expressing neurons given the presence of D2R/OxyR heteromers in the NAc (Bowen and Neumann, 2017), activation of Gq-coupled OxyRs on astrocytes that increase glial transmitter release and restore normal glutamatergic tone on presynaptic glutamate receptors (Moussawi et al., 2011; Dolen et al., 2013), or via the serotonergic system (Cunningham and Anastasio, 2014). Given that micro infusions of supraphysiological levels of OXY directly in the NAc decreased cued reinstatement of cocaine (Weber et al., 2018) and methamphetamine (Baracz et al., 2016; Bernheim et al., 2017; Cox et al., 2017), we were surprised that knocking down the OxyR also prevented cued and drug-primed reinstatement from reaching values beyond extinction responding. Also, NAc shOxyR had no impact on the social interaction task. One thought is that supraphysiological micro-infusion akin to those levels used in previous work also bind vasopressin receptors (Everett et al., 2018). Support for this notion comes from findings that NAc blockade of vasopressin receptors can block OXY’s ability to decrease reinstatement (Everett et al., 2018). These findings suggest that exogenous OXY’s ability to reduce drug seeking in the NAc may not solely rely on its own receptor but may also evoke vasopressin signaling in the NAc. The shOxyR construct we developed was specific for knockdown of the OxyR over vasopressin receptor, so it is possible that endogenous vasopressin signaling during the relapse test contributed to decrease in lever responding during the cocaine-seeking tests. It is possible that OXY binding to vasopressin receptors in the NAc resulted in the null effect on the social interaction task due to the opposing effects of the 2 neuropeptides (Rae et al., 2022). Some aspects of social communication rely on OXY activation of vasopressin (Song et al., 2014) and “cross-talk” between these systems (Song and Albers, 2018), which may have prevented differences in shRNA knockdown in the NAc on social interaction from occurring. However, the complexity of cross-talk between the 2 systems is often task specific, relying on the level of circulating neuropeptide as well as receptor occupancy and availability (Rae et al., 2022). We did not measure OXY peptides in these studies, but it is possible that knockdown of the endogenous OxyR resulted in an increase in the peptide leading to vasopressin receptor binding. The described role of vasopressin combined with endogenous NAc OxyRs knockdown decrease in reinstatement supports the notion that the endogenous action of OxyRs is to promote reinstatement behavior by 1 or more of the mechanisms previously described. Testing this hypothesis and parsing apart the synaptic mechanisms is a crucial future direction in understanding the role of OXY in addiction and other neuropsychiatric disorders.

Knockdown of OxyR in the PFC delayed acquisition of cocaine self-administration, suggesting difficulty acquiring the operant response and or the conditioned associations motivating drug seeking. The PFC is critical for processing information between actions and consequences and consists of neural ensembles that represent task parameter and rules for behavior (Miller, 2000). In the PFC, OxyR-expressing neurons are glutamatergic and GABAergic, 46% and 33%, respectively (Tan et al., 2019). ShOxyR would affect both neural cell types to exert control over behavior potentially through disrupting excitatory and inhibitory balance in the PFC, leading to impairments in executive function. For example, carbetocin (OxyR agonist) infused in the rat PFC decreased fear memory in a passive avoidance task, an effect that was reversed when atosiban (OxyR antagonist) was infused prior (Jang et al., 2022). OXY also improved methamphetamine-induced recognition memory deficits (Fan et al., 2020). And, in human participants with post-synaptic stress disorder, OXY increased working memory and improved cortical connectivity relative to placebo controls (Flanagan et al., 2018). Our findings support the role for OXY action in the PFC in promoting memory, and future studies using manipulations of the endogenous OXY system may elucidate the specific memory functions mediated by OXY.

We also found that PFC shOxyR knockdown produced a seemingly “hyperactive” phenotype during the social interaction task. Importantly, this elevated activity occurred whether or not the caged conspecific was present. shOxyR rats approached the caged conspecific more and also had elevated activity relative to control animals that is consistent with a dysregulated prefrontal cortex (Bubser and Schmidt, 1990; Lacroix et al., 1998) but not necessarily due to differences in social behavior. It is important to note that the animals in this study were individually housed because this variable elevates social behaviors relative to group- or pair-housed rats (Gilles and Polston, 2017; Perkins et al., 2019). The impact of single housing relative to group housing conditions has not been fully investigated regarding endogenous OXY signaling in the PFC. In the paraventricular nucleus of the hypothalamus, however, single-housed animals had fewer OXY cell bodies reported (Gilles and Polston, 2017), with no changes in OXY mRNA expression (Perkins et al., 2019). Given that shCntrl and shOxyR groups were both housed under the same conditions in this study, any impact on housing conditions would affect both groups.

Here, in this report, we offer a glimpse into the complexity of OxyRs in the PFC and NAc in mediating drug intake, reinstatement, and social interaction. In the PFC the role of the receptor conforms to prior literature. That is, intercranial infusions of oxytocin in the PFC increased (Weber et al., 2018) and shRNA knockdown decreased cocaine-cued reinstatement. In contrast, in the NAc, the role OxyRs diverged from the expected pattern because both intercranial infusion (Weber et al., 2018) and shRNA knockdown decreased cocaine-cued reinstatement. The most parsimonious explanation for this pattern is that endogenous NAc OxyRs have a permissive role in relapse that can be overcome by exogenous activation of vasopressin receptors. As molecular tools advance to study the endogenous oxytocin system and identify how OxyR ubiquitous cell type localization affect region and circuit specific function, a better understanding of the system will emerge.

## Data Availability

The data underlying this article will be shared on reasonable request to the corresponding author.

## Interest Statement

The authors do not have any conflicts of interest to report.
